# Aberrant expansion of follicular helper T cell subsets in patients with systemic lupus erythematosus

**DOI:** 10.3389/fimmu.2022.928359

**Published:** 2022-09-02

**Authors:** Xin Jin, Jia Chen, Jian Wu, Ying Lu, Baohua Li, Wenning Fu, Wei Wang, Dawei Cui

**Affiliations:** ^1^ Department of Clinical Laboratory, Tongde Hospital of Zhejiang Province, Hangzhou, China; ^2^ Department of Nephrology Research, The Second Clinical Medical College, Zhejiang Chinese Medical University, Hangzhou, China; ^3^ Department of Clinical Laboratory, Suzhou Hospital Affiliated to Nanjing Medical University, Suzhou, China; ^4^ Department of Rheumatology, Tongde Hospital of Zhejiang Province, Hangzhou, China; ^5^ Department of Blood Transfusion, The First Affiliated Hospital, Zhejiang University School of Medicine, Hangzhou, China

**Keywords:** systemic lupus erythematosus, follicular helper T cells, B cells, plasmablasts, interleukin-21

## Abstract

**Objective:**

Systemic lupus erythematosus (SLE) is a chronic and complex autoimmune disease characterized by multiple autoantibodies, resulting in multiple organ and tissue damages. These pathogenic autoantibodies produced by B cells are closely correlated with follicular helper T (Tfh) cell subsets that play a fundamental role in the pathogenesis of SLE. The aim of the present study was to study the phenotype and role of circulating Tfh (cTfh) cell subsets and associated B cell subpopulations in active and inactive SLE patients.

**Methods:**

Thirty SLE outpatients and 24 healthy controls (HCs) were enrolled in this study. The frequency of cTfh cell and B cell subsets in peripheral blood mononuclear cells (PBMCs) and the plasma levels of eight cytokines were determined by flow cytometry, and plasma IL-21 levels were measured by ELISA. Meanwhile, we used MRL/lpr mice as the model of SLE to research the alterations of Tfh cells in the thymus and spleen of mice.

**Results:**

Frequencies of CD4^+^CXCR5^+^CD45RA^-^effector cTfh cells, PD1^+^cTfh, PD1^+^ICOS^+^cTfh, PD1^+^cTfh1, PD1^+^cTfh2, PD1^+^cTfh17, and PD1^+^ICOS^+^cTfh1 cells as well as plasmablasts showed significant differences among HC, active and inactive SLE patients. Moreover, cytokines typically associated with cTfh cells, including IL-6 and IL-21, were elevated in active SLE patients compared to inactive SLE patients and HCs. Additionally, a positive correlation was observed between PD1^+^ICOS^+^ cTfh or PD1^+^ICOS^+^ cTfh1 cell frequencies and plasmablasts or IL-21 levels, as well as between plasmablasts. We also found PD1^+^ICOS^+^ Tfh cells expansion in both thymus and spleen of MRL/lpr mice, accompanied by increased frequencies in B cells and plasmablasts, meanwhile, cTfh1which expressing IFN-γ was increased in the peripheral blood of MRL/lpr mice.

**Conclusion:**

Tfh cell subsets and plasmablasts may play a fundamental role in the pathogenesis of SLE and may provide potential targets for therapeutic interventions for SLE.

## 1 Introduction

Systemic lupus erythematosus (SLE) is a systemic and complex autoimmune disease characterized by multiple autoantibodies that bind with self-antigens to form immune complexes, which damage multiple organs and tissues (1, 2).Several reports have demonstrated that aberrant antibodies generated by B cells contribute to the development of SLE (3, 4). Previous studies have indicated that the interplay of antigen-presenting cells (APCs), auto-activated CD4^+^T cells and B cells can promote autoantibody and inflammatory cytokine production, which plays an important role in the pathogenesis of SLE (4–6).Several studies have shown that CD4^+^T helper (Th) cell subsets, including Th1, Th2, Th17 and Th22 cells, play critical roles in the immunopathogenesis of SLE (6–8).

Many studies have shown that follicular helper T (Tfh) cells, a subset of CD4^+^T cells located in lymphoid follicles, are different from other Th cells such asTh1, Th2 and Th17 cells (8–11). Tfh cells are characterized by C-X-C motif chemokine receptor 5 (CXCR5), inducible costimulator (ICOS), programmed death-1 (PD-1), high expression of the cytokine interleukin-21 (IL-21), and the involvement of several transcription factors including B cell lymphoma-6 (Bcl-6), signal transducer and activator of transcription-3 (STAT-3) and c-Maf (10, 12–15). Based on the expression of the membrane proteins CXCR3 and chemokine receptor 6 (CCR6), Tfh cells can be divided into three subpopulations: CXCR3^+^CCR6^-^Tfh1, CXCR3^-^CCR6^-^Tfh2, and CXCR3^-^CCR6^+^Tfh17 cells, which share the characteristics of Th1, Th2 and Th17 cells, respectively (9, 16–18). Tfh2 and Tfh17 cells can promote B cell differentiation and autoantibody production and can regulate immunoglobulin (Ig) isotype class switch in many autoimmune diseases including SLE.AlthoughTfh1 cells are commonly considered not to assist B cells, they can also effectively regulate B cell differentiation and induce antibody responses in some vaccine immune responses (16–19).Recent reports have shown that Tfh cells can migrate to C-X-C motif chemokine ligand 13 (CXCL13)-enriched B lymphoid follicles of the germinal center (GC).This promotes GC formation and assist B cells in differentiating into long-living plasma cells that produce high-affinity autoantibodies and mediate autoimmune diseases including SLE (17, 20). Moreover, some reports also demonstrate controversial or conflicting results about the function and role of cTfh cells and their subsets in SLE (20–22). Therefore, the roles of these cTfh cells and their subsets need to be further explored in the pathogenesis of SLE.

In this study, we analyzed the frequencies of effector cTfh cell subsets, including cTfh1, cTfh2, and cTfh17 cells, and their associated cytokines, as well as B cell phenotypes in SLE patients at disease onset. The results showed that the frequencies of effector cTfh including PD1^+^cTfh, PD1^+^cTfh1, PD1^+^cTfh2, PD1^+^cTfh17, PD1^+^ICOS^+^cTfh, and PD1^+^ICOS^+^cTfh1 cells were increased, and the plasmablasts and several associated cytokines were significantly different between active and inactive SLE patients. Additionally, the number of PD1^+^ICOS^+^Tfh cells was expanded in both thymus and spleen of MRL/lpr mice, accompanied by increased frequencies in B cells and plasmablasts. These findings further indicated the important roles of Tfh cells and their subsets in the pathogenesis of SLE.

## 2 Materials and methods

### 2.1 Patient demographics

Thirty outpatients with SLE and twenty-four age- and sex-matched healthy controls (HCs) were enrolled in the study. SLE patients were recruited according to the American College of Rheumatology criteria for the classification of SLE (23), and the exclusion criteria included therapy with immunosuppressive/immunomodulant agents. The SLE Disease Activity Index (SLEDAI) scoring system was used to evaluate clinical disease activity, patients were classified according to having inactive or active disease status: the SLEDAI < 6 group comprised subjects with inactive disease (n=17), while SLEDAI ≧ 6 group consisted of subjects with active disease (n=13). Brief clinical characteristics of the patients with SLE are shown in **Table 1**. This study was approved by the Ethics Committee of Tongde Hospital of Zhejiang Province, and informed consent was obtained from all of the subjects.

**Table 1:** Clinical characteristics of SLE patients and healthy controls (HCs).

Characteristics	SLE active	SLE inactive	HC
Number	13	17	24
Age (years)	38±10	43±13	41±5
Gender (M/F)	2/11	2/15	3/21
SLEDAI active/inactive	13/0	0/17	N
Anti-ANA (+/-)	12/1	3/14	N
ESR (mm/h)	71.3(53–111)	20.6(2–48)	6 (3–15)
CRP (mg/L)	45.6(15.3–120.8)	15.3(2.3–22.1)	2.4 (0–5)
C3 (U/mL)	0.46(0.18–0.74)	0.72(0.49–1.01)	1.32(1.13–1.65)
C4 (U/mL)	0.08(0.02–0.22)	0.21(0.13–0.32)	0.58(0.45–0.61)

SLEDAI, Systemic Lupus Erythematosus Disease Activity Index; M/F, male/female; CRP, C-reactive protein; ESR, erythrocyte sedimentation rate (normal range: men, 0~15 mm/h; women, 0~20 mm/h); C3, complement 3; C4, complement 4 (normal ranges: C3: (0.9~1.8)×10^-3^ U/mL; C4, (0.43~0.64)×10^-3^ U/mL); N, negative; HC: healthy controls.

### 2.2 Animals

Mice Female MRL/MpJ-Fas lpr (MRL-lpr) were purchased from Shanghai SLAC Laboratory Animal Co., Ltd., at 6 weeks of age. Mice MRL-lpr strain as the model for SLE and MRL/MpJ as control. Mice were housed in the laboratory animal research center of Zhejiang Institute of Traditional Chinese Medicine with a 12-h/12-h light/dark, temperature (25 ± 1°C) and humidity (50 ± 5%) environment and an ad libitum supply of food and water. All animal experiments were approved by the Chinese Institutional Animal Care and Use Committee (Approval No. 2019-045). Mice were randomly grouped at eight weeks of age after the onset of disease symptoms to begin the experiment (six mice per cage).

### 2.3 Antibodies and reagents

Following antibodies and isotype-matched antibodies were obtained from Biolegend and Beckman Coulter. Monoclonal antibodies (mAbs) against mouse: FITC-conjugated CD4 (GK1.5, Biolegend), PE-conjugated CD279 (PD-1) (29F.1A12, Biolegend), APC-conjugated CD3 (17A2, Biolegend), PE/Cy7-conjugated CD185 (CXCR5) (L138D7, Biolegend), PB-conjugated CD8a (53-6.7, Biolegend), PE-conjugated CD138 (281-2, Biolegend), APC-conjugated CD19 (6D5, Biolegend), APC-conjugated IFN-γ (XMG1.2, Biolegend), PE/Cy7-conjugated CD278 (ICOS) (71.17G9, Biolegend), PE-conjugated IL-4 (11B11, Biolegend), PerCP/Cy5.5-conjugated IL-17A (TC11-18H10.1, Biolegend); mAbs against human: FITC-conjugated CD183 (CXCR3) (G025H7, Biolegend), PE/Cy5.5-conjugated CD279 (PD-1) (NAT105, Biolegend), PE/Cy7-conjugated CD196 (CCR6) (G034E3, Biolegend), APC-conjugated CD278 (ICOS) (C398.4A, Biolegend), APC Alexa Fluor 750-conjugated CD45RA (2H4LDH11LDB9, Beckman Coulter), BV421-conjugated CD185 (CXCR5) (J252D4, Biolegend), KO-conjugated CD4 (13B8.2, Beckman Coulter), FITC-conjugated CD20 (B9E9, Beckman Coulter), PE-conjugated CD19 (J4.119, Beckman Coulter), PE/Cy5.5-conjugated CD38 (HB-7, Biolegend), APC-conjugated CD27 (M-T271, Biolegend), BV421-conjugated IgD (IA6-2, Biolegend), PE/Cy7-conjugated Foxp3 (259D. Beckman Coulter).

### 2.4 Cell isolation and flow cytometry analysis

Fresh human whole blood was used to isolate peripheral blood mononuclear cells (PBMCs) by density-gradient centrifugation with Ficoll-Hypaque solution (CL5020, CEDARLANE, Canada). Then, the PBMCs were washed three times with phosphate-buffered saline (PBS) and stained with antibodies or istotype-matched controls. In the Mouse, thymus and spleen were ground into single cell suspensions, then stained with antibodies and analyzed by flow cytometry. In human effector cTfh cells were identified as CXCR5^+^CD45RA^-^ cells gated on CD4^+^ T cells, and three cTfh subsets were defined based on CXCR3 and CCR6 as CXCR3^+^CCR6^-^ cTfh1 cells, CXCR3^-^CCR6^-^ cTfh2 cells and CXCR3^-^CCR6^+^ cTfh17 cells (19, 24), follicular regulatory T (Tfr) were identified as CXCR5^+^Foxp3^+^. Plasmablasts were identified as IgD^-^CD38^+^CD20^-^CD27^+^CD19^+^B cells, and class-switched memory B cells were defined as CD38^+^CD20^+^IgD^-^CD27^+^CD19^+^B cells (24, 25). Also, in the analysis of mic, we defined PD1^+^CXCR5^+^ which gated from CD4^+^ T cells as Tfh cells, CD19^+^ as B cells and CD138^+^ as plasma cells. Data acquisition was performed with Navios cytometer (Beckman Coulter, USA). The data were analyzed with FlowJo (Tree Star) software.

### 2.5 Intracellular cytokines in peripheral blood TFH of mice

Heparin-anticoagulated mouse peripheral blood 500ul and 1640 culture medium containing 10% calf serum were mixed, 2ul of stimulant and inhibitor were added respectively (eBioscience Cell Stimulation Cocktail and eBioscience Protein Transport Inhibitor Cocktail, thermofisher) and the cells were incubated for 5 hours at 37°C in 5% CO_2_ environment, then stained for cell surface and intracellular follow the order, and finally detected by flow cytometry, in which IFN-γ expressing CD4^+^CXCR5^+^ cells were defined as cTFH1, IL-4 and IL-17 expressing CD4^+^CXCR5^+^ cells were defined as cTFH2 and cTFH17 respectively.

### 2.6 Immunohistochemistry of mic thymus

Thymuses were extracted from MRL/lpr and MRL/MpJ mice and frozen in OCT compound at -80^°C^ C for 24 hours. Frozen sections of 6 µm were obtained, air dried and fixed in cold acetone for 15 min. The sections were then incubated with a drop of peroxidase blocker for 7 min at room temperature, washed with PBS and incubated with blocking solution for 1 hour. Sections were then incubated with anti-mouse CD19 at 4^°C^ C overnight, followed by incubation with HRP-coupled secondary antibody for 1 hour. Finally, sections were stained with hematoxylin, dehydrated and mounted for observation under an Olympus microscope.

### 2.7 Detection of plasma IL-21

Plasma IL-21 levels in SLE patients and HCs were measured by a human IL-21pre-coatedenzyme-linkedimmunosorbentassay (ELISA) Kit (DAKEWE, China) according to the manufacturer’s instructions. The plasma IL-21 levels were calculated from the standard curve of recombinant human IL-21 cytokine. The brief procedure is as follows: 100 μL plasma was added to sample wells to incubate for 2 hours at room temperature. Then after washing, anti-human IL-21 antibody was added into the wells at 100 μL/well and incubated for 1 hour. Then after washing, HRP solution was added into the wells at 100 μL/well and incubated for 30 minutes at room temperature. After enzyme reaction, *OD* values were measured with an ELISA plate reader, and the concentration was calculated from the standard curve.

### 2.8 Cytometric bead array for plasma cytokines

The levels of eight plasma cytokines (IL-4, IL-10, IL-12, TNF-α, IL-17, IFN-γ, IL-2, IL-6) in SLE patients and HCs were measured by a human Cytometric Bead Array (CBA) Kit (BD) with flow cytometry according to the manufacturer’s instructions. Briefly, 25 µL of each component (the plasma, buffer, Mixed Capture Beads, and biotin-labelled antibody avidin-labelled antibody) was added into the flow tube in order and incubated for 2 hours protected from light. Subsequently, PE-avidin was added and incubated for 30 minutes protected from light, centrifuged to discard supernatant, and 100 µL PBS was added to resuspend the beads. Data were collected by flow cytometry, and the results were analyzed quantitatively using LEGEND plex V8.0 software.

### 2.9 Statistical analysis

All data are expressed as the mean ± standard deviation (SD). GraphPad Prism 9 (GraphPad Software Inc., San Diego, USA) and SPSS 23.0 software (SPSS Inc., Chicago, IL, U.S.A.) were used to analyze the data. Differences between groups were analyzed by multiple t-tests. A *P* value<0.05 was considered to be statistically significant.

## 3 Results

### 3.1 The frequencies of cTfh and Tfr cells in SLE patients

**Figure 1:**
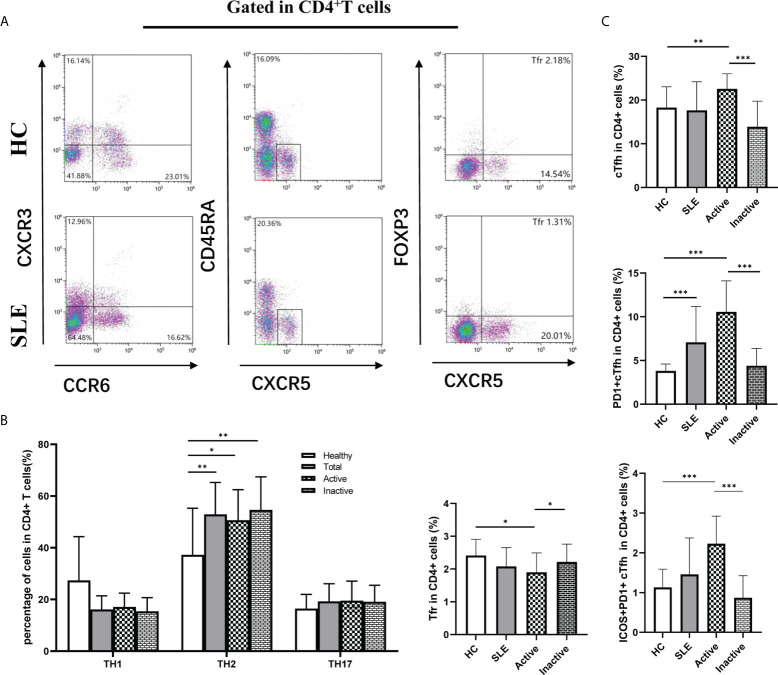
The frequency of effector circulating follicular helper T (cTfh) cells, follicular regulatory T (Tfr) and other Th cells in SLE patients by flow cytometry. **(A)**, Analysis of cTfh cells and other Th cell frequencies in peripheral blood mononuclear cells (PBMCs) by flow cytometry; **(B)**, Percentage of T helper (Th) cells: Th1 (CXCR3^+^CCR6^-^), Th2 (CXCR3^-^CCR6^-^) and Th17 (CXCR3^-^CCR6^+^); **(C)**, Percentage of Tfr, cTfh cells and subsets in CD4^+^ T cells in SLE patients, Tfr: CD4^+^CXCR5^+^FOXP3^+^, Effector cTfh cells: CD4^+^CXCR5^+^CD45RA^-^, based on characterized surface molecules PD1^+^ and ICOS^+^, we define two cTfh subsets, including PD1^+^cTfh and PD1^+^ICOS^+^cTfh. Horizontal lines show the median. Statistically significant differences are indicated by the *P* value, *P<0.05;**P<0.01;***P<0.001.

Circulating Tfh (cTfh) cells play an important role in the pathogenesis of autoimmune diseases. To determine the potential role of effector cTfh cells in SLE patients, thirty SLE patients, including 17 inactive and 13 active patients and 24 age- and sex-matched HCs, were enrolled in this study (**Table 1**). The frequency of cTfh cells in total CD4^+^T cells from SLE patients was analyzed by flow cytometry (**Figure 1A**). In comparison with the HC group, the frequency of CXCR3^-^CCR6^-^Th2 cells (but not CXCR3^+^CCR6^-^Th1 and CXCR3^-^CCR6^+^Th17 cells) was markedly increased in active and inactive SLE patients (**Figure 1B**). The frequencies of effector cTfh cells and ICOS^+^PD-1^+^cTfh cells in CD4^+^T cells were not significantly different between SLE patients and HCs. Further analysis showed that the frequencies of cTfh cells and ICOS^+^PD-1^+^cTfh cells in active SLE patients were higher than those in inactive SLE patients and HCs. Additionally, the frequency of PD1^+^cTfh cells in CD4^+^T cells was remarkably increased in active SLE patients in comparison with HCs, and a significant difference was also observed between active and inactive SLE patients. We also found the proportion of Tfr cells was lower in active group compared to HCs and inactive patient groups (**Figure 1C**).

### 3.2 Alterations of cTfh cell subsets in SLE patients

**Figure 2:**
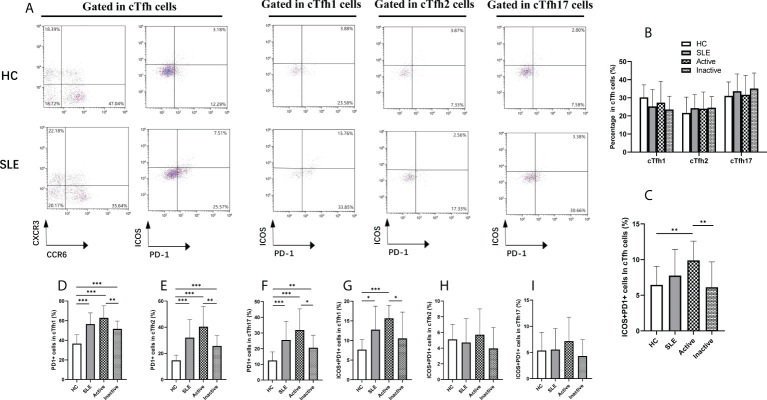
Changes of cTfh cell subsets in SLE patients. **(A)**, Analysis of cTfh cell subsets by flow cytometry; **(B)**, Percentage of cTfh1, cTfh2 and cTfh17 cells in cTfh cells from SLE patients: cTfh1 (CXCR3^+^CCR6^-^ cTfh), cTfh2 (CXCR3^-^CCR6^-^ cTfh) and cTfh17 (CXCR3^-^CCR6^+^ cTfh); **(C)**, Percentage of ICOS^+^PD-1^+^ cTfh cells in cTfh cells from SLE patients; **(D–F)**, Percentage of PD-1^+^ cTfh1, PD-1^+^ cTfh2, PD-1^+^ cTfh17 cells in cTfh cells from SLE patients; **(G–I)**, Percentage of ICOS^+^PD-1^+^ cTfh1, ICOS^+^PD-1^+^ cTfh2, ICOS^+^PD-1^+^ cTfh17 cells in cTfh cells from SLE patients. Horizontal lines show the median. Statistically significant differences are indicated by the *P* value, *P<0.05;**P<0.01;***P<0.001.

Based on CXCR3 and CCR6 expression on cTfh cells, cTfh cell are categorized into three subtypes: CXCR3^+^CCR6^-^cTfh1, CXCR3^-^CCR6^-^cTfh2, and CXCR3^-^CCR6^+^cTfh17 cells, which were detected by flow cytometry in this study (**Figure 2A**). The frequencies of cTfh1, cTfh2 and cTfh17 cells in cTfh cells were not significantly different among the active SLE, inactive SLE, and HC groups (**Figure 2B**). However, the PD1^+^ICOS^+^cTfh cell frequencies in cTfh cells were significantly elevated in active SLE patients compared to inactive SLE patients and HCs (**Figure 2C**). Moreover, the frequency of PD1^+^cTfh1 cells in cTfh1 cells was significantly higher in active SLE patients than in HCs and inactive SLE patients (**Figure 2D**). Similarly, the frequencies of PD-1^+^cTfh2 cells in cTfh2 cells and PD-1^+^cTfh17 cells in cTfh17 cells differed significantly among HC, active SLE, and inactive SLE groups (**Figures 2E, F**). Additionally, the ratio of PD1^+^ICOS^+^cTfh1 cells in cTfh1 cells (but not PD1^+^ICOS^+^cTfh2 cells in cTfh2 cells and PD1^+^ICOS^+^cTfh17 cells in cTfh17 cells) was significantly higher in active patients than in HCs and inactive SLE patients (**Figures 2G–I**).

### 3.3 The frequencies of B cell subsets in SLE patients

**Figure 3:**
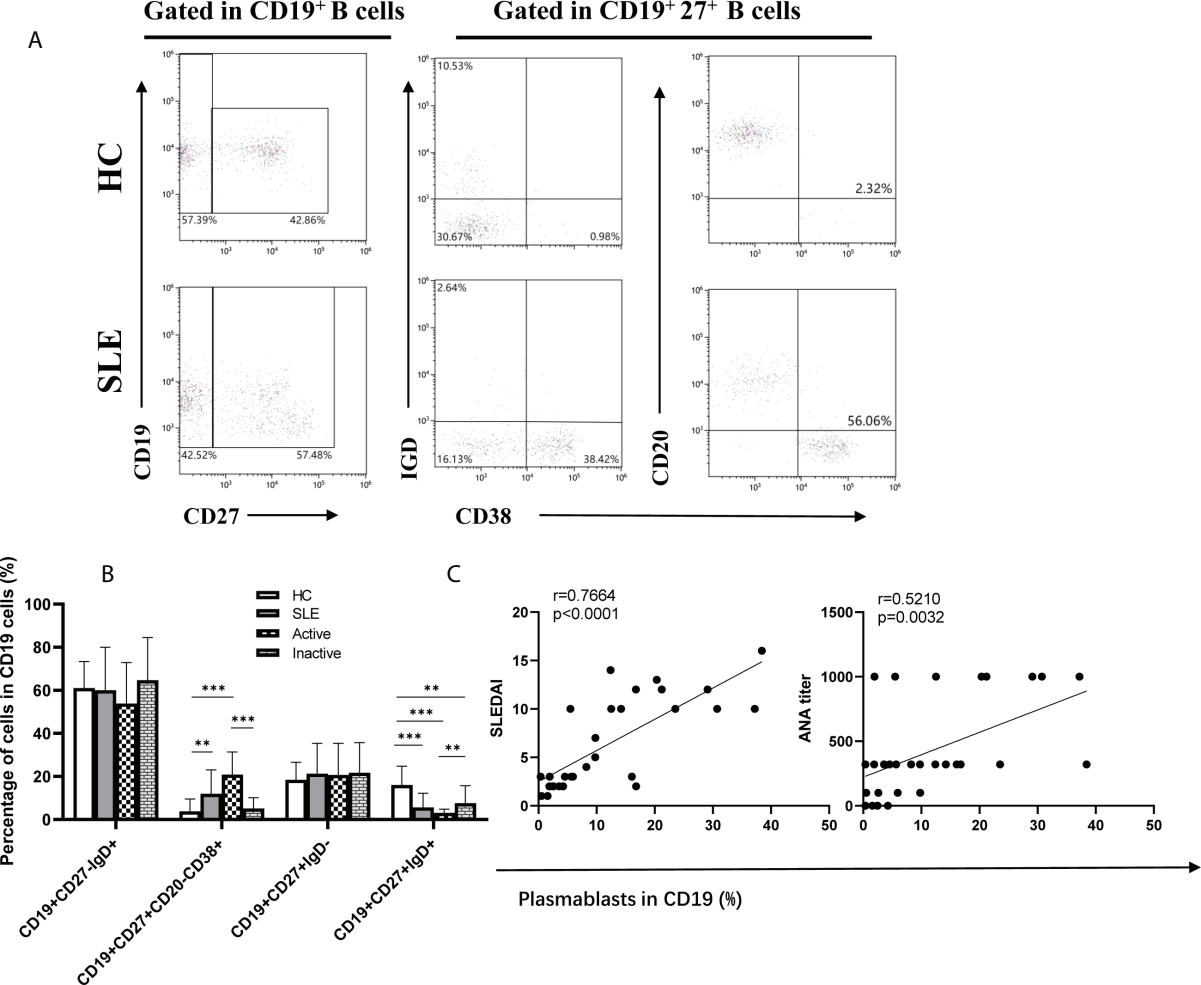
Frequencies of B cell subsets in SLE patients. **(A)**, Analysis of B cell subsets by flow cytometry; **(B)**, Percentage of naïve B cells (CD19^+^CD27^-^IgD^+^), plasmablasts (CD19^+^CD27^+^CD20^-^CD38^+^), class-switched memory B cells (CD19^+^CD27^+^IgD^-^), and IgM memory B cells (CD19^+^CD27^+^IgD^+^) in SLE patients; **(C)**, The correlations between percentage of Plasmablast and SLEDAI, ANA titer. Horizontal lines show the median. Statistically significant differences are indicated by the *P* value, *P<0.05;**P<0.01;***P<0.001.

Assisted by Tfh cells, B cells play critical roles in the occurrence and progression of SLE. Therefore, B cell subsets were analyzed by flow cytometry (**Figure 3A**). In comparison with HCs, the frequency of plasmablasts (CD19^+^CD27^+^CD20^-^CD38^+^) was dramatically increased in SLE patients, particularly in active SLE patients, but it was not noticeably different between inactive patients and HCs (**Figure 3B**). Conversely, the frequencies of IgM memory B cells (CD19^+^CD27^+^IgD^+^), class-switched memory B cells (CD19^+^CD27^+^IgD^-^), and naïve B cells (CD19^+^CD27^-^IgD^+^) differed significantly among the active, inactive SLE patients and HC groups (**Figure 3B**), and interestingly, the frequencies of plasmablast were positively associated with SLEDAI score (r=0.7664, p<0.0001), anti-ANA titers (r=0.5210, p=0.0032) (**Figure 3C**).

### 3.4 Plasma cytokine levels in SLE patients

**Figure 4:**
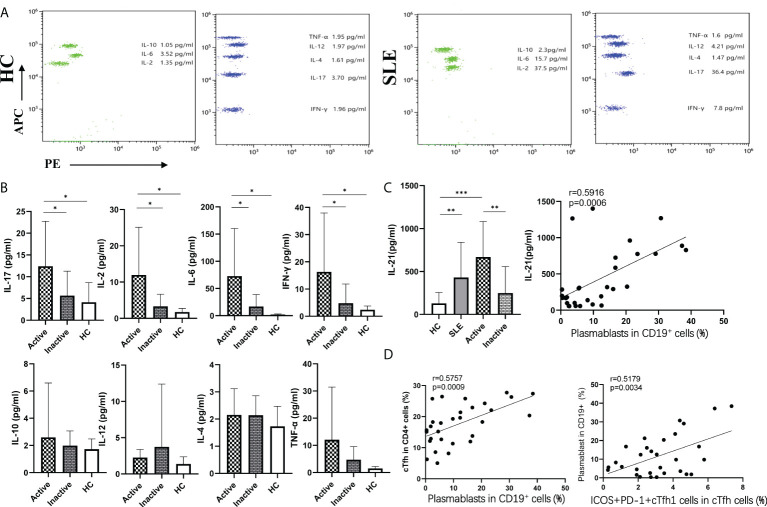
Plasma cytokine levels in SLE patients. **(A)**, Cytokine levels in plasma from SLE patients and HCs by flow cytometry; **(B)**, The levels of eight cytokines were detected by flow cytometry; **(C)**, The levels of plasma IL-21were detected by ELISA in SLE patients and HCs; **(D)**, Relationship between important indicators in SLE patients. *p < 0 05, **p < 0 01, and ***p < 0 001.

To explore the potential role of plasma cytokines in cTfh cell frequencies, the plasma levels of eight cytokines (IL-4, IL-10, IL-12, TNF-α, IL-17, IFN-γ, IL-2, and IL-6) in SLE patients and HCs were measured by a human CBA Kit using flow cytometry (**Figure 4A**), and plasma IL-21 levels were tested by ELISA in SLE patients and HCs. The results showed that the plasma levels of the cytokines IL-17, IL-6, IL-2, IL-21 and IFN-γ were remarkably increased in active SLE patients compared to inactive SLE and HC groups, but there were no significant differences between the inactive SLE and HC groups. However, plasma levels of IL-4, IL-10, IL-12, and TNF-α were not significantly different among active, inactive patients and HCs (**Figure 4B**). Moreover, plasma IL-17 and IL-2 concentrations were positively correlated with SLEDAI score (IL-17: r=0.3811, p=0.0377; IL-2: r =0.3865, p=0.0349) (**Supplementary Materials**).

### 3.5 Analysis of the relationship among important indicators in SLE patients

Interestingly, the frequencies of plasmablasts were positively associated with cTfh cell frequencies (r=0.5757, p=0.0009), PD1^+^ICOS^+^cTfh cell frequencies in CD4^+^T cells (r=0.6315, p=0.0002), SLEDAI scores (r=0.7664, p<0.0001), IL-21 levels (r=0.5918, p=0.0006), and ICOS^+^PD1^+^cTfh1 cell frequencies in cTfh cells (r=0.5179, p=0.0034). Moreover, the frequencies of PD1^+^ICOS^+^cTfh cells in CD4^+^T cells were positively correlated with plasma IL-21 levels (r=0.5080, p=0.0042), which were also positively correlated with ICOS^+^PD1^+^cTfh1 cells (r=0.4239, p=0.0196) (**Figures 4C, D**).

### 3.6 Alterations of immune cells in the MRL/lpr mice

**Figure 5:**
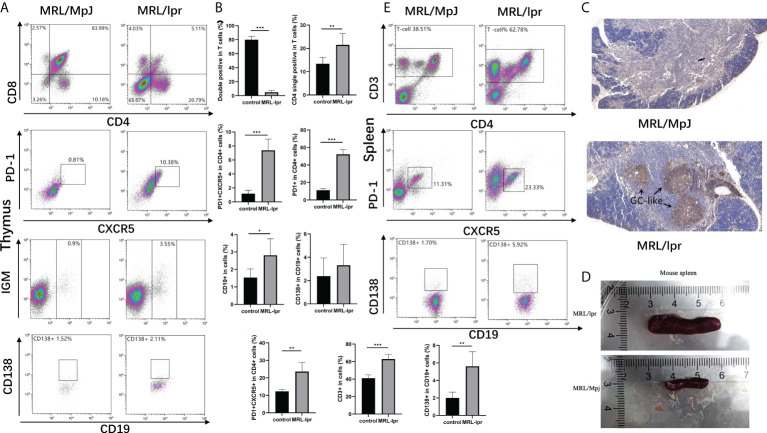
Alterations of immune cells in the MRL/lpr mice. **(A)**, Analysis of immune cell in the thymus of mice; **(B)**, Percentage of double positive cells (CD8^+^CD4^+^), CD4 single positive cells (CD4^+^), Tfh cells (CXCR5^+^PD-1^+^), B cells (CD19^+^), and Plasma cells (CD138^+^) in the thymus of MRL/lpr mice (n = 6 for each group). **(C)**, Representative images of CD19 staining of thymic tissue from MRL/lpr mice and control. Scale bar: 100μm. **(D)**, Spleens were enlarged in MRL/lpr mice when compared with age- and sex-matched MRL/MpJ mice. **(E)**, Analysis of immune cell in the spleen of mice. F, Percentage of T cells (CD3^+^), Tfh cells (CXCR5^+^PD-1^+^), B cells (CD19^+^), and Plasma cells (CD138^+^) in the spleen of MRL/lpr mice (n = 6 for each group). *P<0.05; **P<0.01; ***P<0.001.

In order to explore whether T cells alter during the generation and maturation in SLE patients, we researched the composition of immune cells in the thymus using MRL/lpr mice as a model, as results showed, in the thymus of MRL/lpr mice, the proportion of double positive cells (CD8^+^CD4^+^) decreased and the CD4 single positive cells as well as PD1^+^CXCR5^+^ Tfh cells increased. Meanwhile, there were also more B cells in the thymus of model mice, histochemistry showed a large accumulation of B cells in the thymus of diseased mice, as well as the formation of germinal centers-like structures. but no difference was found between the two groups for CD138^+^ plasmablasts (**Figures 5A–C**). We also found an expansion of T cells and PD1^+^CXCR5^+^ Tfh cells in the spleen of MRL/lpr mice and an increase in the proportion of plasmablasts compared to control (**Figures 5D–F**).

### 3.7 Aberrant expansion of peripheral blood cTfh subsets in the MRL/lpr mice

**Figure 6:**
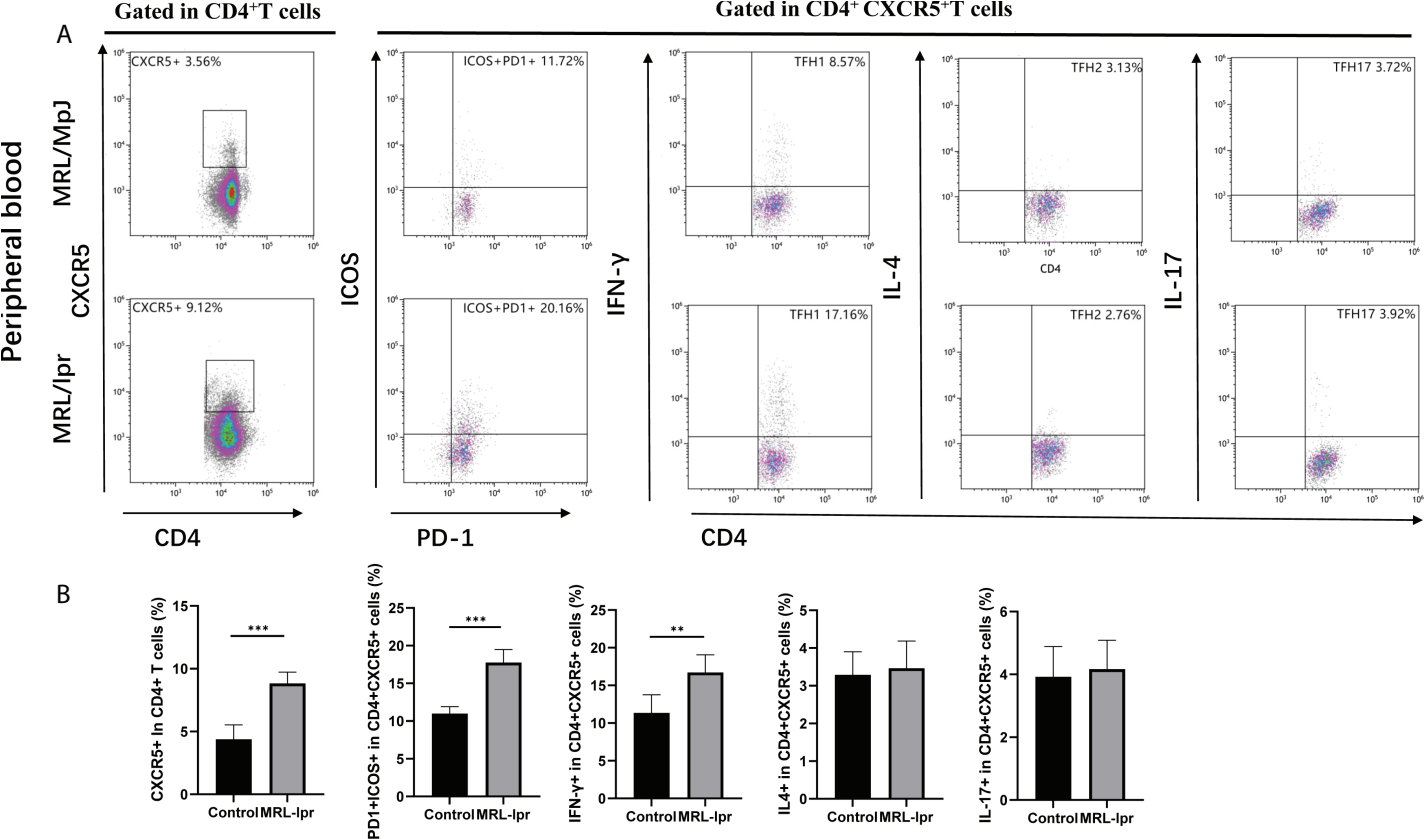
Aberrant expansion of peripheral blood cTfh subsets in the MRL/lpr mice. **(A)**, Analysis of cTfh cell subsets in the peripheral blood of mice; **(B)**, Percentage of cTfh cells (CXCR5^+^CD4^+^), ICOS positive cTfh cells (ICOS^+^PD-1^+^CXCR5^+^CD4^+^), cTfh1 cells (IFN- γ^+^CXCR5^+^CD4^+^), cTfh2 cells (IL-4^+^CXCR5^+^CD4^+^), and cTfh17 cells (IL-17^+^CXCR5^+^CD4^+^) in the peripheral blood of MRL/lpr mice (n = 6 for each group). *P<0.05; **P<0.01; ***P<0.001.

Comparing peripheral blood cTfh cells in MRL/lpr and control mice, we found that CXCR5^+^CD4^+^ cTfh cells and PD1^+^ICOS^+^ CXCR5^+^CD4^+^ cTfh subset were significantly higher in MRL/lpr mice than in control. Also, when comparing cTfh subset based on secreting different cytokines, the results showed that IFN-γ expressing cTfh1 were significantly higher in MRL/lpr mice, while IL-4 and IL-17 expressing cTfh2 and cTfh17 cells were not found to be significantly different in the two groups (**Figures 6A, B**).

## 4 Discussion

Circulating Tfh (cTfh) cells, contain long-lived memory cells characterized by CD4^+^CXCR5^+^CD45RA^-^ in peripheral blood, are functionally similar to GC Tfh cells, can induce autoantibody production by B cells, and play an important role in the pathogenesis of autoimmune diseases including SLE (19–23, 25, 26). In this study, we found that the proportion of Th2, effector cTfh, PD1^+^cTfh, and PD1^+^ICOS^+^cTfh cells was significantly higher in active SLE patients than in HCs and inactive SLE patients, but these cTfh cell frequencies were not significantly different between inactive SLE patients and HCs, which was consistent with previous studies (20, 21). Moreover, our results showed that the frequencies of plasmablasts were closely correlated with the frequencies of cTfh cells, ICOS^+^PD-1^+^cTfh cells in CD4^+^T cells, anti-ANA titers and SLEDAI scores in SLE patients (20, 22). An increased plasmablast population can produce autoantibodies, including anti-ANA antibodies, and correlates with disease activity, which plays critical roles in the pathogenesis of SLE (27, 28). Previous reports showed that dysregulation of cTfh cells could help naïve B cells differentiate into plasmablasts to produce autoantibodies and was related to the development of SLE (10, 29, 30). Additionally, the frequencies of PD1^+^ICOS^+^Tfh cells, B cells and plasmablasts were significantly increased in both thymus and spleen of MRL/lpr mice, respectively, which were accorded with previous reports (27, 28). These findings imply the relationship of these Tfh cells and the disease activity of SLE to some extent, and that Tfh cells and their subsets play important roles in the development of SLE.

Based on the expression of CCR6 and CXCR3, cTfh cells are divided into three major subsets: cTfh1, cTfh2 and cTfh17 cells (19, 25). cTfh2 and cTfh17 cells are more effective than cTfh1 cells in inducing the differentiation of B cells to produce antibodies (22, 25). Previous reports showed that increased frequencies of cTfh2 and/or cTfh 17 cells and a decrease in cTfh1 cells were observed in SLE patients and mice, and were associated with autoantibody titers and disease activity (25, 31, 32). However, several studies have shown that an elevated frequency of cTfh1 cells is observed in active SLE patients, and that an increase in ICOS^+^PD-1^+^cTfh1 cell population is correlated with the number of plasmablasts and high-affinity antibody production in humans after influenza vaccines (22, 33, 34). Our results showed that no significant difference was observed in the frequencies of cTfh1, cTfh2 and cTfh17 cells among HCs, active and inactive SLE patients. Interestingly, the proportion of PD-1^+^cells incTfh1, cTfh2 and cTfh17 cells was remarkably increased in active and inactive SLE patients compared to HCs, and only ICOS^+^PD-1^+^cTfh1 cell (but not ICOS^+^PD-1^+^cTfh2 and ICOS^+^PD-1^+^cTfh17 cell) frequencies were significantly increased in active SLE patients and correlated with plasmablasts number, meanwhile, we also found cTfh1which expressing IFN-γ was increased in the peripheral blood of MRL/lpr mice. These findings suggested an important role of cTfh cell subsets in the development of SLE, and the discrepancy of cTfh cell subsets from other studies might reflect the number of SLE patients and the criteria of the SLEDAI score in the present study.

Cytokine-skewed Tfh cells can regulate B differentiation, antibody production and Ig isotype class-witching, which play critical roles in the humoral immune response of autoimmune diseases including SLE (19, 35). Conventional Tfh cell differentiation require IL-6 and IL-21,which induce naïve CD4^+^T cells to differentiate into Tfh cells by activating STAT-3 and upregulating Bcl-6 (10, 19). Moreover, IL-21 produced primarily by Tfh cells can also drive B cells to differentiate into plasmablasts and produce antibodies (29, 30, 36). Our results suggested that plasma levels of IL-6 and IL-21 were significantly higher in active SLE patients than in HCs and inactive SLE patients, and plasma IL-21 levels were positively correlated with plasmablasts and activated ICOS^+^PD-1^+^cTfh cells in SLE patients. These findings suggested the important role of IL-6 and IL-21 in regulating the humoral immune response in the pathogenesis of SLE. Additionally, our results demonstrated high levels of IL-2, IL-17 and IFN-γ in plasma from SLE patients, particularly in active SLE patients, but plasma levels of TNF-α, IL-4, IL-10 and IL-12 were not different among HC and SLE patients, which was partly consistent with previous reports (19, 33, 34, 37). Differentiated Tfh cells can also produce high levels of IL-2 that can negatively inhibit Tfh cell differentiation by activating the transcription factor STAT-5, which contributes to maintain the balance of Tfh cells *in vivo (* 19, 35). IL-12 can lead to Th1 and Tfh1 cell differentiation by regulating T-bet and Bcl-6 signals to secrete IFN-γ and enhance antibody isotype class-switching (19, 33). The differentiation of Tfh2 cells is dependent on IL-4, which is also self-secreted by Tfh2 cells and can contribute to humoral immune responses, and IL-17 from Tfh17 cells can also promote antibody production and class-switching (19, 38–40). These results indicate that SLE is a complex autoimmune disease characterized by dysfunction of cTfh cell subsets and disorder of Tfh-associated cytokines.

Our study currently has some limitations. First, the total number of specimens from outpatients with SLE, especially those with active SLE, is limited because the cohort of active SLE patients without drug therapy at the time of study enrolment is small. Several studies have demonstrated that drug treatments can affect cTfh cell frequencies (23, 31, 41). Therefore, a larger cohort will be investigated to clarify the role of cTfh cell subsets in SLE patients in the future. Additionally, the function of cTfh cells and their subsets that help B cell differentiation will be validated by co-culture experiments of cTfh subsets and B cells from SLE patients. Furthermore, due to too few cells after stimulation, the failure to perform further subset analysis of cTfh1, cTfh2 and cTfh17 in MRL/lpr mice based on the expression of PD1 and ICOS is also a limitation of this study, and modified animal experiments should be performed to explore the role of Tfh cells and their subsets in lupus mice in the future.

## 5 Conclusion

In conclusion, we found an increased proportion of the PD-1^+^cTfh1, PD-1^+^cTfh2, PD-1^+^cTfh17, and ICOS^+^PD-1^+^cTfh1 cell subsets in SLE patients, which was associated with the disease activity of SLE patients. Moreover, our further results showed the increased number of PD1^+^ICOS^+^Tfh cells, B cells and plasmablasts in both thymus and spleen, CTfh1 cells in peripheral blood of MRL/lpr mice. Taken together, our results provide novel insights into the role of cTfh cell subsets in SLE patients, which may contribute to the development of new biological targets for therapeutic interventions for SLE.

## Data availability statement

The original contributions presented in the study are included in the article/**Supplementary Material**. Further inquiries can be directed to the corresponding authors.

## Ethics statement

The studies involving human participants were reviewed and approved by the ethics committee of Tongde Hospital of Zhejiang Province. The patients/participants provided their written informed consent to participate in this study. The animal study was reviewed and approved by Ethics Committee of Tongde Hospital of Zhejiang Province. Written informed consent was obtained from the individual(s) for the publication of any potentially identifiable images or data included in this article (Identification Nos. 2019045).

## Author contributions

XJ and DC drafted the manuscript and designed the figures and tables. WF, YL, and BL collected samples from the SLE patients. JC, XJ, and YL performed the experiments. JW and WW revised the manuscript. DC, JW, and WW conceived the topic. All authors contributed to the article and approved the submitted version.

## Funding

This work was supported by the National Natural Science Foundation of China (81871709), National Natural Science Foundation of China (81974549), and the Natural Science Foundation of Zhejiang Province, China (LY16H200001).

## Acknowledgments

We appreciate the highly qualified native English speaking editors at American Journal Experts (AJE) for providing an excellent English language editing service (verification code: A562-CD48-A02A-A633-4F14) for our manuscript.

## Conflict of interest

The authors declare that the research was conducted in the absence of any commercial or financial relationships that could be construed as a potential conflict of interest.

## Publisher’s Note

All claims expressed in this article are solely those of the authors and do not necessarily represent those of their affiliated organizations, or those of the publisher, the editors and the reviewers. Any product that may be evaluated in this article, or claim that may be made by its manufacturer, is not guaranteed or endorsed by the publisher.

## Supplementary material

Supplementary Figure 1 Correlation analyze between the SLEDAI and the concentration of IL-17, IFN-γ, IL-2, IL-6 in lupus patients (n=30).
