# Supplementary figures and images for: Aberrant expansion of follicular helper T cell subsets in patients with systemic lupus erythematosus
Source: Front Immunol. 2022 Sep 2;13:928359. doi: 10.3389/fimmu.2022.928359 (PMC9478104; doi:10.3389/fimmu.2022.928359)

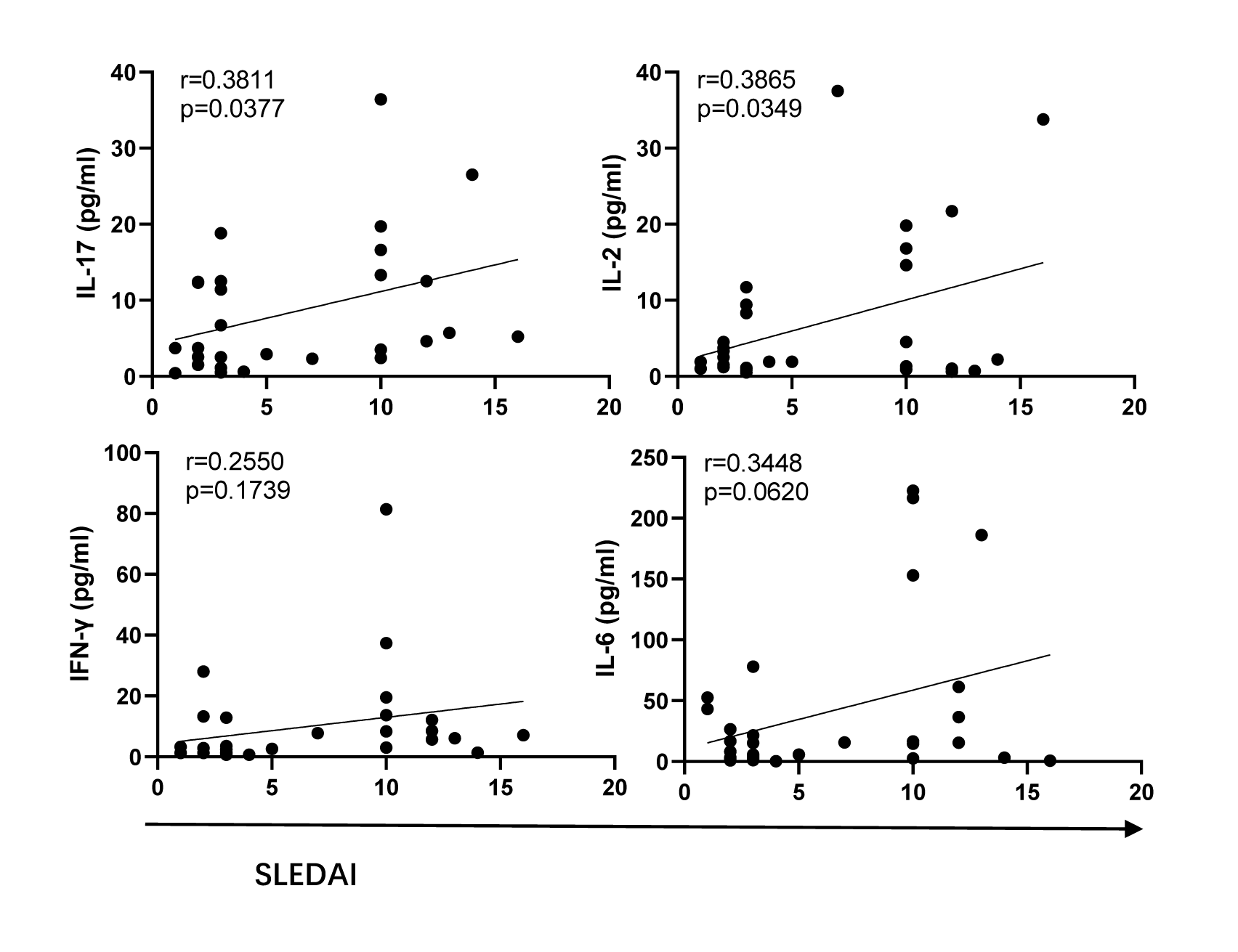

Supplement: Supplementary Figure 1 — Correlation analyze between the SLEDAI and the concentration of IL-17, IFN-γ, IL-2, IL-6 in lupus patients (n=30). [file Image_1.tif]
